# Does Allocation of Attention Influence Relative Velocity and Strength of Illusory Line Motion?

**DOI:** 10.3389/fpsyg.2018.00147

**Published:** 2018-02-22

**Authors:** Timothy L. Hubbard, Susan E. Ruppel

**Affiliations:** ^1^Department of Psychology, Arizona State University, Tempe, AZ, United States; ^2^Department of Psychology, University of South Carolina Upstate, Spartanburg, SC, United States

**Keywords:** illusory line motion, attention, perception of velocity, perception of motion, cueing

## Abstract

In illusory line motion, presentation of a cue is followed by presentation of a nearby stationary line, and the line is perceived to “unfold,” “expand,” or “extend” away from the cue. Effects of the allocation of attention regarding where the cue or the line would be presented were measured in three experiments, and ratings of relative velocity and relative strength of illusory motion were collected. Findings included (a) relative velocity and relative strength decreased with increases in SOA from 50 to 450 ms, (b) relative velocity and relative strength were not influenced by whether illusory motion moved from one end of the line to the other or from both ends toward the middle of the line, (c) increased uncertainty regarding where the line would appear did not influence relative velocity or relative strength, and (d) valid pre-cues regarding the location of a cue resulted in faster relative velocity than did invalid pre-cues, but pre-cue validity did not influence relative strength. Implications of these findings for the relationship of such illusory motion and attention (e.g., divided attention, shifts in attended location) are considered.

## Introduction

In illusory line motion (ILM; also referred to as the *line motion effect*, the *line-motion illusion, motion induction*, and the *shooting line illusion*), a specific location is cued (e.g., by having a stimulus appear or vanish, Hikosaka et al., 1993a), and shortly thereafter, a stationary line appears near the cued location. The entirety of the line is presented simultaneously, but observers perceive the line to be presented sequentially such that the line is perceived to “unfold” or “be drawn” from the near end of the line (i.e., the end closest to the cued location) to the far end of the line (i.e., the end farthest from the cued location). Even though there is no actual motion in the display, observers report the far end of the line is perceived as moving away from the cued location and the line extends or expands from the near end to the far end. The experiments reported here examined effects of the allocation of attention on this illusory motion. Allocation of attention was manipulated by presenting one or two cues, by varying the predictability of whether the line appeared to the left or to the right of a cue, by varying whether the line appeared relatively near or far from the cue, and by providing a valid or invalid verbal pre-cue regarding where the cue would appear. Participants rated the relative velocity and the relative strength of illusory motion, and how these ratings were influenced by where the cue or the line could appear was considered.

The initial studies of ILM induced illusory motion by a luminance change at one of two potential cue locations, and a line then appeared between those locations; this has been referred to as _flash_ILM by Hamm (2017). ILM-like effects can be induced by similarities in color (e.g., if potential cues are objects of different colors and the line matches the color of the actual cue, referred to as _color_ILM, e.g., von Grünau and Faubert, 1994) or shape (e.g., if potential cues are objects of different heights and the line matches the height of the actual cue, referred to as _shape_ILM, e.g., Corballis et al., 2002). Ha et al. (2017; Hamm, 2017) suggested these examples of illusory motion do not reflect a unitary phenomenon and that _color_ILM and _shape_ILM are examples of transformational apparent motion (TAM; Tse, 2006) and would be more appropriately referred to as _color_TAM and _shape_TAM. Similarly, displays in which a single cue location is presented and then a line appears have been suggested to reflect polarized gamma motion (PGM; Kanizsa, 1979) rather than ILM *per se* (Hamm, 2017). Han et al. (2016) suggested attempts to find a single mechanism to account for all types of ILM-like effects, or attempts to rule out a given mechanism based on failure of that mechanism to account for one type of ILM-like effect, are misguided. Consistent with this, Hamm (2017) suggested _flash_ILM resulted from effects of attention and _color_TAM and _shape_TAM resulted from effects of attribute priming (cf. Faubert and von Grünau, 1995). Given the similar phenomenology of _flash_ILM, _color_TAM, _shape_TAM, and PGM, such illusory motions will be collectively referred to as ILM-like effects.

The earliest accounts of ILM-like effects were based on _flash_ILM and suggested an ILM-like effect resulted from an attentional gradient (e.g., Hikosaka et al., 1993a,b; Shimojo et al., 1999). If an observer allocates more attention to a cued location, then less attention is available for processing stimulus information farther from that location. Portions of the line closer to the cued location thus receive more attention than do portions of the line farther from the cued location; given that stimuli in attended locations are processed more quickly than are stimuli in unattended locations (e.g., Hawkins et al., 1990; Talsma et al., 2007), portions of the line nearer the cued location enter perceptual awareness more quickly than do portions of the line farther from the cued location. This results in a perception that the line unfolds from the cued location. Indeed, _flash_ILM activates neural areas involved with visual attention and motion processing (Hamm et al., 2014). However, Hamm and Klein (2002) found response times to targets at the far end of a line that exhibited _flash_ILM did not differ from response times to targets at the cued location, and they suggested any differences in attention occurred after illusory motion. In their study, the primary task of participants was to judge or detect a stimulus other than the line; it is possible that effects of attention on illusory motion might be more robust if the primary task of participants involved judging the line (e.g., velocity or strength of illusory motion) rather than judging a stimulus other than the line.

Many accounts and studies of ILM-like effects do not explicitly address perceived velocity of illusory motion, although several studies used matching or cancelation of motion of an actually moving line as an investigative tool (e.g., von Grünau et al., 1996b; Fuller and Carrasco, 2009; Han et al., 2016; Ha et al., 2017). In a study in which a stationary line was presented, Hubbard and Ruppel (2011) had participants rate relative velocities of illusory motion in which distance between the cue and the near end or far end of line varied or line length varied; they found that increases in distance did not influence ratings of relative velocity, but increases in line length did result in ratings of faster relative velocity. The relationship of velocity of illusory motion to strength of illusory motion in ILM-like effects has not been extensively investigated, but Christie and Klein (2005) suggested the sense of illusory motion should be stronger with slower apparent drawing speeds (i.e., an inverse relationship with perceived relative velocity). Based on Christie and Klein (2005), Hubbard and Ruppel (2011) suggested a more extreme rating of direction indicated a stronger sense of motion, and although intuitively appealing, this idea confounded strength of direction with strength of motion and did not yield completely consistent results. Therefore, it might be useful to separate judgments of motion strength from judgments of motion direction.

In the experiments reported here, participants viewed displays based on _flash_ILM (two potential cues, one at each end of the subsequent line) and PGM (a single cue and a line) displays previously used to study ILM-like effects. The experiments were designed to investigate the relationship between attention and ILM-like effects, and allocation of attention regarding the location of the cue and the location of the line were varied. Experiment 1 presented one cue on the left side or right side of the display, cues on both the left side and right side of the display, or no cue. Experiment 2 presented a cue on the left side, in the center, or on the right side of the display, and the line was presented in the left-center or right-center of the display. Experiment 3 presented a verbal (printed) pre-cue instructing participants whether the upcoming cue would be on the left side or right side of the display, and the pre-cue was valid on 80% of the trials and invalid on 20% of the trials. The amount of attention allocated to the cued location(s), and the number of potential directions of illusory motion from a cue and distance of the line from the cue, varied across experimental conditions. In all experiments, ratings of relative velocity of (illusory) motion and ratings of relative strength of (illusory) motion were collected. Differences in ratings of relative velocity and ratings of relative strength across different experimental conditions should provide insight into the properties of ILM-like effects, the role of attention in ILM-like effects, and potential constraints for theories of ILM-like effects.

## Experiment 1

**FIGURE 1:**
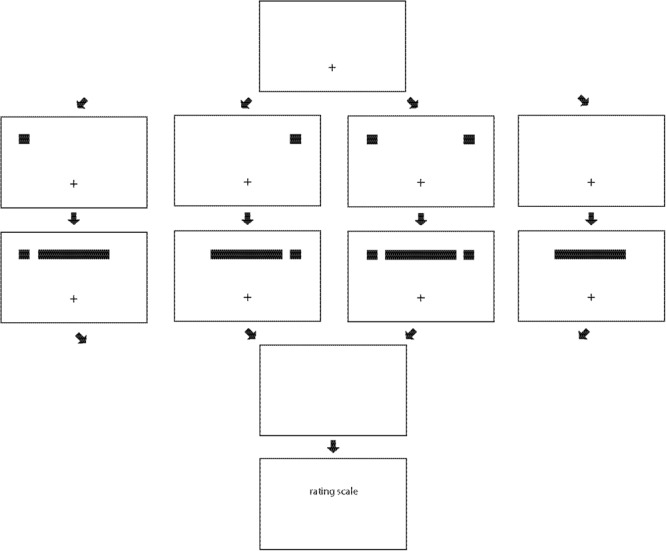
The structure of trials in Experiment 1. A fixation point is presented **(first/top row)**, then a cue appears in the upper left, in the upper right, in the upper left and in the upper right, or no cue appears **(second row)**. A line is then presented **(third row)**, the cue(s) (if present) and line vanish **(fourth row)**, and then the rating scale appears **(fifth/bottom row)**.

Experiment 1 presented displays similar to those used in previous studies of ILM-like effects (e.g., Hikosaka et al., 1993a; Downing and Treisman, 1997; Schmidt, 2000), and a schematic is shown in **Figure 1**. A fixation point appeared horizontally centered in the bottom half of the display. A cue was then presented in the upper left, the upper right, the upper left and the upper right, or no cue was presented. If one cue was presented, more attention could be allocated to that cue than if attention was divided between two spatially separated cues. A stationary horizontal line then appeared (equivalent to the ILM_con_ condition in Han et al., 2016), and after a brief delay, the cue(s) (if present) and the line vanished. Previous studies found that if one cue was presented, illusory motion was usually from the end of the line closest to the cue toward the end of the line most distant from the cue (e.g., Hikosaka et al., 1993a; Schmidt, 2000; although see Hikosaka et al., 1993b; Crawford et al., 2006), and if cues were presented at each end of the line simultaneously, illusory motion was usually from each end of the line toward the middle of the line (e.g., Faubert and von Grünau, 1992; Downing and Treisman, 1997). However, whether the relative velocity or the relative strength of illusory motion if two cues are present (and attention is divided) differs from the relative velocity or the relative strength of illusory motion if one cue is present (and attention is not divided), and how such differences might be influenced by SOA, is not known.

### Method

#### Participants

The participants in all experiments were undergraduates at the University of South Carolina Upstate, who received partial course credit and were naïve to the hypotheses. The procedures in all experiments were approved by the Institutional Review Board at the University of South Carolina and were in accordance with the Declaration of Helsinki, and all participants provided written informed consent prior to participation. Sixteen undergraduates participated in Experiment 1.

#### Apparatus

The stimuli were displayed upon and the data collected with a Gateway desktop computer equipped with a 15-inch color monitor with a refresh rate of 60 Hz and a resolution of 1024 × 768 pixels. Participants’ head and eye movements were not constrained, and the average viewing distance was approximately 60 cm.

#### Stimuli

The cue was a black square 20 pixels in width and in height (∼0.83°), and the line was a black rectangle 196 pixels in width and 20 pixels in height (∼8.13 × 0.83°); the cue and the line (1.9 cd/m^2^) were presented on a white background (103 cd/m^2^). The line was located slightly above the vertical midpoint of the display, vertically aligned with the cue(s) (if present), and horizontally centered in the display. The right edge of the left cue and left edge of the right cue were each 120 pixels (∼4.98°) from the vertical axis of the display (and 372 pixels [∼15.44°] from the left and right edges of the display), respectively. There was a separation of 20 pixels (∼0.83°) of empty space between the cue and the line. The SOA between appearance of the cue and appearance of the line was 50, 250, or 450 ms. The fixation point was a plus shape, and each arm of the plus shape was 10 pixels in length (i.e., the plus shape was 20 pixels in width and 20 pixels in height). The fixation point was located at the horizontal center of the display and approximately one-third of the vertical distance from the bottom to the top of the display.

Ratings of relative velocity were made using a 1–7 scale in which 1 was “very slow” and 7 was “very fast.” Ratings of relative strength were made using a 1–7 scale in which 1 was “very weak” and 7 was “very strong.” In one block of trials, participants rated relative velocity of (illusory) motion, and in another block of trials, participants rated relative strength of (illusory) motion^1^. In each block, each participant received 48 trials (4 [cue: left, right, both, none] × 3 [SOA: 50, 250, 450 ms] × 4 replications) in a different random order.

#### Procedure

Rating task was blocked, with ratings of relative velocity collected in one block and ratings of relative strength collected in another block. Order of blocks was counterbalanced across participants. Before beginning each block, participants were given a practice session consisting of 10 practice trials randomly drawn from experimental trials for that block. When participants were ready for a trial to begin, they pressed a designated key. The cue(s) (if present) appeared after a delay of 250 ms, and the line appeared after an additional 50, 250, or 450 ms. The cue(s) (if present) and the line were visible for an additional 250 ms, and then the cue(s) (if present) and the line vanished. After a 250 ms pause, a rating scale appeared centered in the display and remained visible until the participant responded. Ratings were entered by pressing the appropriate key on a numeric keypad. After participants entered their ratings, the display cleared, and participants were prompted to begin the next trial.

### Results

**FIGURE 2:**
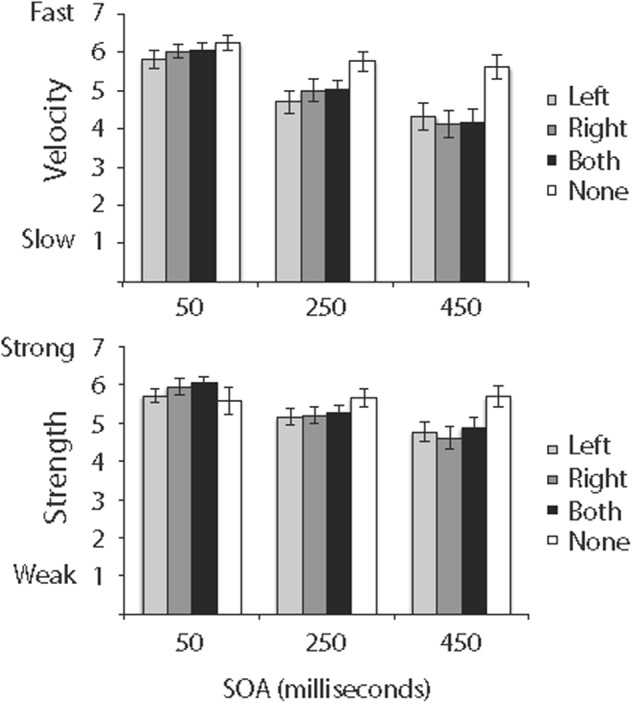
Ratings of the relative velocity **(top)** and relative strength **(bottom)** of ILM in Experiment 1. Error bars reflect standard error of the mean.

Ratings of relative velocity are shown in the top panel of **Figure 2**, and ratings of relative strength are shown in the bottom panel of **Figure 2**.

#### Velocity

Ratings of relative velocity were analyzed in a 4 (cue) × 3 (SOA) repeated measures ANOVA^2^. Cue, *F*(3,45) = 12.12, *MSE* = 0.73, *p* < 0.001, SOA, *F*(2,30) = 15.77, *MSE* = 2.27, *p* < 0.0001, and Cue × SOA, *F*(6,90) = 3.72, *MSE* = 0.40, *p* < 0.003 were significant. As shown in the top panel of **Figure 2**, if one or two cues were presented, ratings of relative velocity decreased as SOA increased, and if no cue was presented, ratings of relative velocity were not influenced by SOA (even if a cue was not presented, the SOA manipulation still resulted in differences in the latency between when a trial was initiated and when the line was presented). Additionally, least squares comparisons of cues revealed ratings of relative velocity if no cue was presented (*M* = 5.88, *SE* = 0.15) were significantly different from ratings if one cue was on the left (*M* = 4.94, *SE* = 0.20), if one cue was on the right (*M* = 5.05, *SE* = 0.20), or if cues were on the left and right (*M* = 5.08, *SE* = 0.19). Also, least squares comparisons of SOAs revealed all pairwise comparisons between 50-ms (*M* = 6.04, *SE* = 0.11), 250-ms (*M* = 5.12, *SE* = 0.15), and 450-ms (*M* = 4.55, *SE* = 0.19) SOAs were significant, although the significant Cue × SOA interaction in the top panel of **Figure 2** suggests those differences were driven by conditions in which at least one cue was present.

#### Strength

Ratings of relative strength were analyzed in a 4 (cue) × 3 (SOA) repeated measures ANOVA. Cue, *F*(3,45) = 2.81, *MSE* = 0.65, *p* < 0.05, SOA, *F*(2,30) = 11.38, *MSE* = 1.01, *p* < 0.0001, and Cue × SOA, *F*(6,90) = 5.09, *MSE* = 0.34, *p* < 0.001, were significant. As shown in the bottom panel of **Figure 2**, if one or two cues were presented, ratings of relative strength decreased as SOA increased, and if no cue was presented, ratings of relative strength were not influenced by SOA. Additionally, least squares comparisons of cues revealed ratings if no cue was presented (*M* = 5.64, *SE* = 0.17) were significantly different from ratings if a cue was on the left (*M* = 5.21, *SE* = 0.14) or if a cue was on the right (*M* = 5.26, *SE* = 0.16) but did not differ from ratings if cues were on the left and right (*M* = 5.40, *SE* = 0.15). Also, least squares comparisons of SOAs revealed all pairwise comparisons between 50-ms (*M* = 5.82, *SE* = 0.12), 250-ms (*M* = 5.34, *SE* = 0.11), and 450-ms (*M* = 4.98, *SE* = 0.15) SOAs were significant, although the significant Cue × SOA interaction in the bottom panel of **Figure 2** suggests those differences were driven by conditions in which at least one cue was present.

### Discussion

If one or two cues were presented, then ratings of relative velocity of illusory motion decreased with increases in SOA between appearance of the cue(s) and appearance of the line. This pattern extends the effect of SOA on ratings of relative velocity reported for single-cue stimuli in Hubbard and Ruppel (2011) to include presentation of two cues and illusory motion from both ends of the line to the middle of the line. Interestingly, velocity ratings were not influenced by whether one cue or two cues were presented, and this suggests the time required for the entire line to unfold if two cues were presented (and illusory motion was from both ends of the line toward the middle of the line) was half that of the time required for the entire line to unfold if one cue was presented (and illusory motion was from one end of the line toward the opposite end of the line). Hubbard and Ruppel (2011) found that ratings of perceived velocity increased with increases in line length; given that the extent (i.e., portion or percentage) of the line to be traversed from a given cue if one cue was presented was twice the extent of the line to be traversed from a given cue if two cues were presented, it could have been predicted that relative velocity if one cue was presented would have been faster than relative velocity if two cues were presented. Why relative velocity appears to be influenced by line length (Hubbard and Ruppel, 2011), but not influenced by the portion of the line to be traversed (Experiment 1), is not yet clear.

If one or two cues were presented, then ratings of relative strength of illusory motion decreased with increases in SOA between appearance of the cue(s) and appearance of the line. The decreases in ratings of relative velocity and in ratings of relative strength with increases in SOA do not appear consistent with Christie and Klein’s (2005) suggestion that a stronger sense of illusory motion is related to a slower drawing speed, nor does the decrease in ratings of relative strength with increases in SOA appear consistent with Hubbard and Ruppel’s (2011) suggestion that relative strength increases with increases in SOA. In Christie and Klein and in Hubbard and Ruppel, relative strength of illusory motion was inferred on the basis of the extremity of ratings on a scale in which endpoints of the scale were defined as motion to the left or right and midpoint of the scale was defined as no motion. It appears that more extreme ratings of direction in Christie and Klein and in Hubbard and Ruppel might not unequivocally indicate stronger ILM-like effects. Rather, it appears that an illusory motion is perceived as stronger if that illusory motion occurs at a faster velocity; one possible explanation is that faster targets are perceived as possessing more momentum (cf. effects of velocity on representational momentum, Hubbard, 2005), and because it takes more effort to stop an object that possesses more momentum, faster motion is perceived as stronger.

The none trials (in which no cue was presented) were included as a control condition in Experiment 1, and as expected, participants usually chose the option “appeared all at once” when indicating direction (see Footnote 1). As might be expected, ratings of velocity and ratings of strength in the none condition were not influenced by SOA, whereas ratings of velocity and ratings of strength when one or two cues were presented decreased with increases in SOA (resulting in significant Cue × SOA interactions). It might seem odd to have participants rate velocity or strength of motion if no cue was presented, but none trials were randomly intermixed with trials in which one or two cues were presented, and this allowed an *a priori* possibility of perceived motion on each trial. Surprisingly, ratings of velocity or strength if a cue was not presented were significantly higher than ratings of velocity or strength if one or two cues were presented. One possibility is that occurrence of illusory motion on 75% of the trials (left, right, both) biased participants to expect motion, and so simultaneous appearance of the entirety of the line in none trials was interpreted as a faster velocity or stronger motion. Such a possibility is intuitive for ratings of velocity, because if velocity of unfolding was faster, then the entirety of the line would have appeared more quickly. Such a possibility is not as intuitive for ratings of strength, unless a stimulus that appeared all at once was judged as making a stronger entrance than a stimulus that gradually appeared.

## Experiment 2

**FIGURE 3:**
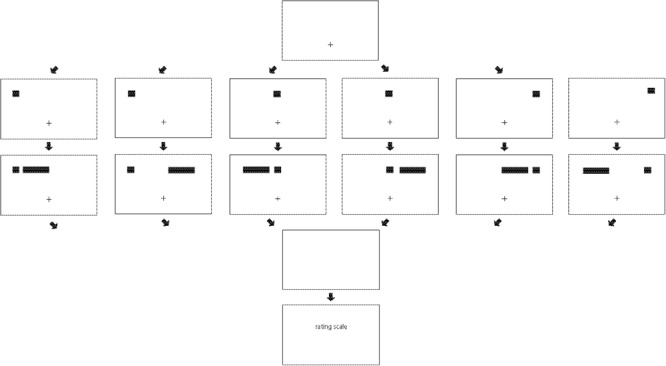
The structure of trials in Experiment 2. A fixation point is presented **(first/top row)**, then a cue appears in the upper left, in the upper center, or in the upper right **(second row)**. A line is then presented in the left-center or right-center **(third row)**. The cue and line vanish **(fourth row)**, and then the rating scale appears **(fifth/bottom row)**.

In Experiment 1, the line always appeared in the same location. It is not clear whether a similar pattern of relative velocities or relative strengths of illusory motion would occur if there were more uncertainty regarding where the line would appear. Accordingly, and as shown in **Figure 3**, Experiment 2 presented a cue on the left side, in the center, or on the right side of the display. After a brief delay, a horizontal line was presented in the left-center (right of a left cue and left of a center cue or a right cue) or in the right-center (left of a right cue and right of a center cue or a left cue) of the display. Given that a left cue and a right cue could only be followed by lines to the right or left, respectively (i.e., one potential direction from the cue), but a center cue could be followed by a line to the right or by a line to the left (i.e., two potential directions from the cue), expectations regarding the specific direction to the line from the cue should be stronger for left cues and right cues than for center cues. Thus, it might be predicted that any attentional gradient would be stronger with decreases in the number of potential directions in which the line could appear (i.e., stronger for the cues on the left or right [one possible direction] than for cues in the center [two possible directions], cf. the fan effect, Anderson and Reder, 1999). Although it might appear intuitive that illusory motion for a line farther from the cue would be rated as weaker and slower, Hubbard and Ruppel (2011) reported that distance of the line from the cue did not influence ratings; thus, an effect of distance of the line from the cue is not predicted.

### Method

#### Participants

The participants were 18 undergraduates from the same participant pool as in Experiment 1, and none had participated in that experiment.

#### Apparatus

The apparatus was the same as in Experiment 1.

#### Stimuli

The fixation point, cues, and lines were the same as in Experiment 1, with the following exceptions: The center cue was located on the vertical axis of the display, and edges of the left cue and right cue closest to the vertical axis were 230 pixels (∼9.55°) to the left and right of the vertical axis, respectively. The left-center line and right-center line were each 180 pixels (7.47°) in length, and the line length was shortened slightly from the line length used in Experiment 1 so the spatial coordinates of the ends of the left-center line and the spatial coordinates of the ends of the right-center line could be constant regardless of the location of the cue. The distance between the cue and the line was 20 pixels (a left cue and a left-center line, a right cue and a right-center line, a center cue and a left-center or right-center line) or 260 pixels (∼10.79°; a left cue and a right-center line, a right cue and left-center line). The rating scales were the same as in Experiment 1. In one block of trials, participants rated relative velocity of (illusory) motion, and in another block of trials, participants rated relative strength of (illusory) motion. In each block, each participant received 72 trials (3 [cue: left, center, right] × 2 [line: left-center, right-center] × 3 [SOA: 50, 250, 450 ms] × 4 replications) in a different random order.

#### Procedure

The procedure was the same as in trials in which one cue was presented in Experiment 1.

### Results

**FIGURE 4:**
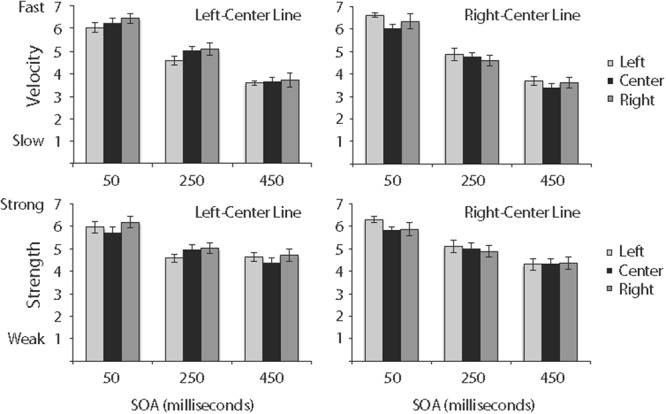
Ratings of the relative velocity **(top)** and relative strength **(bottom)** of ILM in Experiment 2. Data for lines in the left-center are in the left column, and data for lines in the right-center are in the right column. Error bars reflect standard error of the mean.

Ratings of relative velocity are shown in the top panel of **Figure 4**, and ratings of relative strength are shown in the bottom panel of **Figure 4**.

#### Velocity

Ratings of relative velocity were analyzed in a 3 (cue) × 2 (line) × 3 (SOA) repeated measures ANOVA. SOA was significant, *F*(2,34) = 88.95, *MSE* = 2.16, *p* < 0.0001, and least squares comparisons revealed all pairwise comparisons between 50-ms (*M* = 6.27, *SE* = 0.08), 250-ms (*M* = 4.81, *SE* = 0.09), and 450-ms (*M* = 3.61, *SE* = 0.11) SOAs were significant. No other main effects or interactions were significant.

#### Strength

Ratings of relative strength were analyzed in a 3 (cue) × 2 (line) × 3 (SOA) repeated measures ANOVA. SOA was significant, *F*(2,34) = 51.27, *MSE* = 1.27, *p* < 0.0001, and interacted with Line, *F*(2,34) = 6.59, *MSE* = 0.17, *p* < 0.01. As shown in the bottom panel of **Figure 4**, differences between relative strength of ILM of the left-center line and relative strength of ILM of the right-center line were slightly greater with a 250-ms SOA than with 50- or 450-ms SOAs. Additionally, least squares comparisons revealed all pairwise comparisons between 50-ms (*M* = 5.98, *SE* = 0.09), 250-ms (*M* = 4.92, *SE* = 0.09), and 450-ms (*M* = 4.45, *SE* = 0.11) SOAs were significant. No other main effects or interactions were significant.

### Discussion

As in Experiment 1, ratings of relative velocity and ratings of relative strength decreased with increases in SOA. Ratings of relative velocity and ratings of relative strength were not influenced by whether the line was in the left-center or the right-center of the display, and lack of Cue × Line interactions for ratings of relative velocity and for ratings of relative strength suggests the distance of the line from the cue (e.g., whether a left cue preceded a left-center line or a right-center line) did not generally influence relative velocity or relative strength of illusory motion. This is consistent with the lack of an effect of distance of the near end or the far end of the line from the cue in ratings of relative velocity in Hubbard and Ruppel (2011). Ratings of relative velocity and ratings of relative strength were not influenced by whether the cue was on the left, center, or right of the display. The similarity of ratings of relative velocity to ratings of relative strength, regardless of the location of the cue, suggests that increased uncertainty regarding whether the line would appear to the left or right of the cue (that occurred with the center cue relative to the left cue or the right cue) does not influence relative velocity or relative strength of illusory motion. However, even though uncertainty regarding where the line would be presented did not appear to influence illusory motion, it is possible that uncertainty regarding where the cue would appear could influence illusory motion, and this was examined in Experiment 3.

## Experiment 3

**FIGURE 5:**
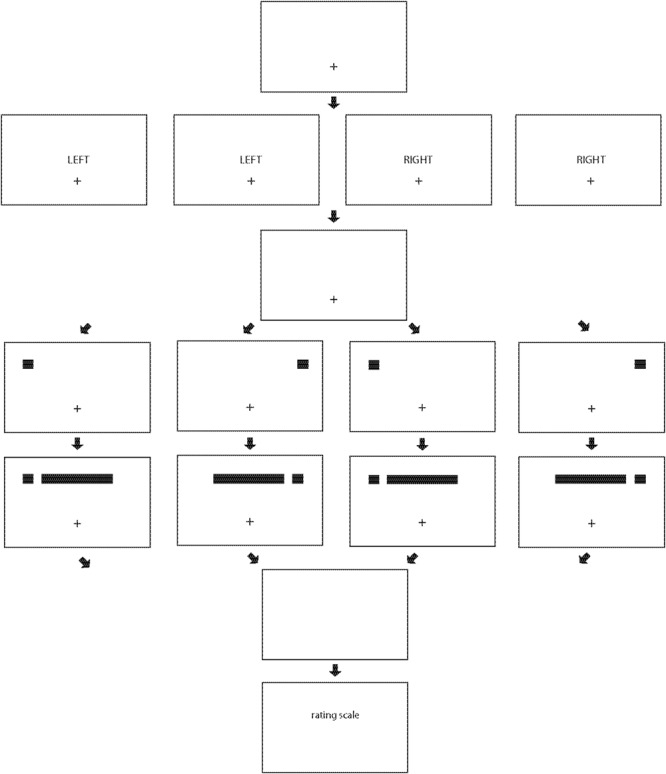
The structure of trials in Experiment 3. A fixation point is presented **(first/top row)**. A pre-cue (the word “LEFT” or “RIGHT” then appears **(second row)**. The pre-cue vanishes **(third row)**. A cue appears in the upper left or in the upper right **(fourth row)**. A line is then presented **(fifth row)**. The cue and line vanish **(sixth row)**, and then the rating scale appears **(seventh/bottom row)**.

As noted by Han et al. (2016), the attentional gradient hypothesis requires that attention be focused at a specific location prior to the appearance of the line. In previous studies of ILM-like effects, unless participants happened to be attending to the location where the cue appeared (which was unlikely, given the use of a fixation point in many studies), appearance of the cue triggered a shift of attention to the location of the cue. Such a shift would require a minimal amount of time, and this might influence subsequent illusory motion if the shift was not completed prior to appearance of the line. Accordingly, and as shown in **Figure 5**, Experiment 3 presented a verbal pre-cue (the printed word “LEFT” or “RIGHT”) to inform participants whether the cue would appear on the left side or right side of the display, and the pre-cue was valid (e.g., the pre-cue suggested the cue would appear on the left, and the cue appeared on the left) on 80% of the trials and invalid (e.g., the pre-cue suggested the cue would appear on the left, and the cue appeared on the right) on 20% of the trials. If the pre-cue was valid, then no additional shift of attention to the cued location would be necessary when the cue appeared, but if the pre-cue was invalid, then an additional shift of attention to the cued location would be necessary when the cue appeared. A greater allocation of attention at the cued location in the valid cue condition than in the invalid cue condition when the line appeared could result in a stronger attentional gradient along the line, and this might influence relative velocity and relative strength of illusory motion.

### Method

#### Participants

The participants were 15 undergraduates from the same participant pool used in Experiment 1, and none had participated in Experiments 1 or 2.

#### Apparatus

The apparatus was the same as in Experiment 1.

#### Stimuli

The fixation point, cues, and lines were the same as in Experiment 1, with the following exceptions: One cue (either on the left or the right) was presented on each trial. The pre-cue consisted of the printed word “LEFT” or “RIGHT” and was presented in 24 pt. font black text and located in the center of the display. The rating scales were the same as in Experiment 1. In one block of trials, participants rated relative velocity of (illusory) motion, and in another block of trials, participants rated relative strength of (illusory) motion. In each block, each participant received 48 trials (2 [cue: left, right] × 2 [validity: valid pre-cue, invalid pre-cue] × 3 [SOA: 50, 250, 450 ms] × 4 replications) in a different random order.

#### Procedure

The procedure was the same as in trials in which one cue was presented in Experiment 1, with the following exceptions: After participants pressed the key to begin a trial, there was a delay of 250 ms before the pre-cue was presented and remained visible for 2 s. The pre-cue then vanished, and after a delay of 250 ms, the cue appeared on the left side or right side of the display.

### Results

**FIGURE 6:**
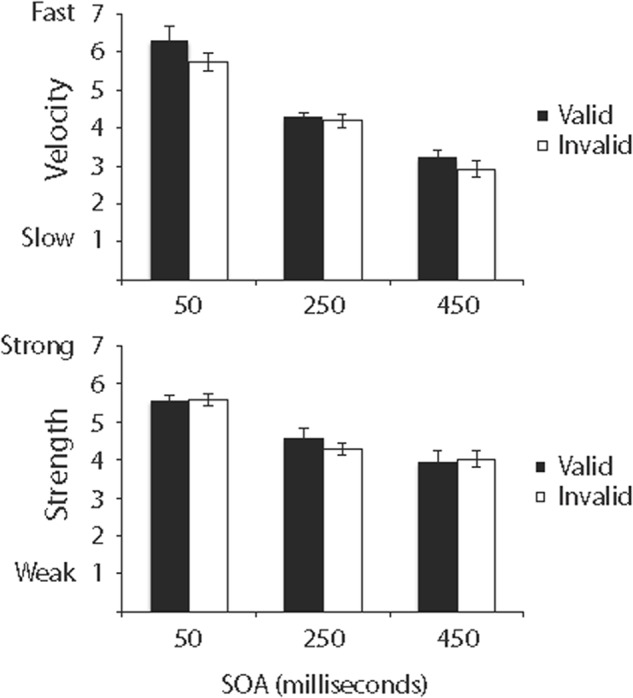
Ratings of the relative velocity **(top)** and relative strength **(bottom)** of ILM in Experiment 3. Error bars reflect standard error of the mean.

Ratings of relative velocity are shown in the top panel of **Figure 6**, and ratings of relative strength are shown in the bottom panel of **Figure 6**.

#### Velocity

Ratings of relative velocity were analyzed in a 2 (cue) × 2 (validity) × 3 (SOA) repeated measures ANOVA. Validity was significant, *F*(1,14) = 4.97, *MSE* = 0.91, *p* < 0.05, with valid pre-cues (*M* = 4.60, *SE* = 0.20) resulting in ratings of faster relative velocity than did invalid pre-cues, (*M* = 4.28, *SE* = 0.17). SOA was significant, *F*(2,28) = 38.95, *MSE* = 3.39, *p* < 0.001, and least squares comparisons revealed all pairwise comparisons between 50-ms (*M* = 6.01, *SE* = 0.23), 250-ms (*M* = 4.22, *SE* = 0.11), and 450-ms (*M* = 3.07, *SE* = 0.15) SOAs were significant. No other main effects or interactions were significant.

#### Strength

Ratings of relative strength were analyzed in a 2 (cue) × 2 (validity) × 3 (SOA) repeated measures ANOVA. SOA was significant, *F*(2,28) = 13.21, *MSE* = 3.03, *p* < 0.001, and least squares comparisons revealed the 50-ms (*M* = 5.53, *SE* = 0.11) SOA was significantly different from the 250-ms (*M* = 4.42, *SE* = 0.16) and 450-ms (*M* = 3.97, *SE* = 0.19) SOAs, and the 250- and 450-ms SOAs were not significantly different. No other main effects or interactions were significant.

### Discussion

Ratings of relative velocity were influenced by the validity of the pre-cue, such that valid pre-cues resulted in ratings of faster relative velocities than did invalid pre-cues. It is possible that if a valid pre-cue allowed more attention to be focused at the location where the cue would appear, then processing of the nearby portion of the line might be more facilitated, and so relative velocity of illusory motion would be faster. However, ratings of relative strength were not influenced by validity of the pre-cue. Ratings of relative velocity and ratings of relative strength were dissociated, and this suggests (a) velocity of ILM-like effects is not determined solely by strength of illusory motion and (b) strength of ILM-like effects is not determined solely by velocity of illusory motion. It might be that if a given threshold is exceeded, then the relative strength of illusory motion in an ILM-like effect doesn’t increase further even if velocity is increased. With the benefit of additional activation from a valid pre-cue, that threshold was exceeded in the valid pre-cue condition but not in the invalid pre-cue condition or in Experiments 1 and 2. The effects of the pre-cue are consistent with claims that endogenous attention can influence ILM-like effects (e.g., Schmidt, 2000; Chica et al., 2008) but inconsistent with claims that endogenous attention does not influence ILM-like effects (e.g., Ishigami et al., 2009; Christie, 2014). Also, and consistent with Experiments 1 and 2, ratings of relative velocity and ratings of relative strength decreased with increases in SOA.

It might be suggested that if attention was allocated to the location specified by the pre-cue, then any illusory motion should have been away from the pre-cue location (regardless of validity), as the pre-cue would have established a gradient of attention which was highest at the pre-cued location. Alternatively, illusory motion from the pre-cue location in invalid trials might have canceled out illusory motion from the subsequent exogenous physical cue, thus eliminating any illusory motion in invalid pre-cue trials. However, presentation of an invalid pre-cue did not reverse or cancel out illusory motion, and examination of participants’ responses regarding direction (see Footnote 1) indicated illusory motion in all conditions was perceived to precede from the cued location. It is unlikely the effect of an invalid pre-cue would be as strong or stronger than the effect of the cue at the time the line appeared. Even if the initial effect of an invalid pre-cue was as strong as the initial effect of the cue, the greater latency between the pre-cue and line than between the cue and line would suggest attention was greater at the cued location than at the invalid pre-cue location at the time the line appeared. The greater activation at the cued location than at the invalid pre-cue location resulted in illusory motion away from the cued location, but with less velocity than when all attention had been allocated to the cued location. However, why the difference between valid pre-cues and invalid pre-cues influenced ratings of velocity but not ratings of strength is not clear.

It is interesting to compare the results of Experiment 3 to the results of Ha et al. (2017), who incorporated _flash_ILM trials within a larger set of experimental trials that also contained target detection trials. At the beginning of each trial, Ha et al.’s (2017) participants viewed a digital numeral 8 on the left side and on the right side of the display. Either the digit on the left side or on the right side would flash (i.e., function as a cue), and the participants would have to respond if either (a) one of the 8s changed to a digital 2 or 5 or (b) a line connecting the two digital 8s appeared to expand rightward from the digit on the left, appeared all at once, or appeared to expand leftward from the digit on the right. Ha et al. (2017) varied validity of the cue in target detection trials; the magnitude of the costs and benefits of cueing on target detection (of a 2 or 5) were correlated positively with the magnitude of _flash_ILM, and Ha et al. (2017) suggested cueing and _flash_ILM were mediated by a common mechanism. Such a correlation is consistent with the effect of cue validity on ratings of relative velocity in Experiment 3, as well as with previous findings regarding a role for exogenous attention in _flash_ILM (e.g., Christie and Klein, 2005; Christie, 2014) and with the attentional gradient hypothesis. A role for attention in accounting for the data of Experiment 3 does not, of course, suggest that exogenous attention or an attentional gradient is necessarily involved in all ILM-like effects.

It is also interesting to compare the results of Experiment 3 to the results of Chica et al. (2008), who presented a modified _flash_ILM display in which one of two circles was cued and a line then appeared between the circles. A dot was superimposed on the end of the line nearest the cue or the end of the line farthest from the cue, and participants had to detect the dot (i.e., identify at which end of the line the dot appeared) or discriminate the color of the dot. In a block of cued trials, the dot was adjacent to the cued circle on 75% of the trials, and in a block of uncued trials, the dot was adjacent to the uncued circle on 75% of the trials. The cueing manipulation in Chica et al. (2008) is similar to the validity manipulation of pre-cues in Experiment 3. The finding of greatest relevance in Chica et al. (2008) for Experiment 3 is that cueing modulated the strength of _flash_ILM if participants discriminated the color of the dot but not if participants merely detected the dot. As Experiment 3 did not involve a discrimination task, the trials in Experiment 3 are more similar to the detection task than to the discrimination task in Chica et al. (2008), and the lack of a difference in ratings of strength for valid trials and for invalid trials in Experiment 3 is consistent with the lack of a difference in ratings of strength of _flash_ILM between cued trials and uncued trials in the detection task in Chica et al. (2008). The results of Chica et al. (2008) and of Experiment 3 are consistent with the hypothesis that endogenous information can modulate ILM-like effects.

## General Discussion

Two empirical findings consistent across Experiments 1, 2, and 3 are that (a) ratings of relative velocity of illusory motion decreased with increases in SOA from 50 to 450 ms, and (b) ratings of relative strength of illusory motion decreased with increases in SOA from 50 to 450 ms. Additionally, three empirical findings specific to Experiments 1, 2, and 3, respectively, are that (a) relative velocity of illusory motion was not influenced by whether one cue or two cues were presented, (b) increased uncertainty regarding the location where the line would appear (after the cue was presented but before the line was presented) did not influence relative velocity or relative strength of illusory motion, and (c) ratings of relative velocity, but not ratings of relative strength, were higher if participants were presented with a valid pre-cue that indicated the location where the cue would appear than if participants were presented with an invalid pre-cue that indicated a location different from where the cue would appear. These findings have implications for understanding potential contributions of the allocation of attention and expectation to ILM-like effects and for understanding the relationship between relative velocity and relative strength of ILM-like effects. Surprisingly, and with the exception of the valid pre-cue condition in Experiment 3, manipulation of the allocation of attention did not influence ratings of relative velocity or of relative strength of illusory motion.

One issue regarding the relationship between attention and ILM-like effects is whether such effects are influenced by whether attention is divided or undivided. The similarity of ratings of relative velocity to ratings of relative strength when one or two cues were presented in Experiment 1, and the similarity of ratings of relative strength to ratings of relative velocity when the location of a cue was predictive of only one potential direction or predictive of two potential directions of ILM in Experiment 2, suggests ILM-like effects are not influenced by whether attention is divided or undivided. One hypothesis is that similarities of ratings when one cue was presented to ratings when two cues were presented (in Experiment 1) might have resulted from participants attending to only one cue regardless of whether one or two cues were presented, but such a hypothesis is not consistent with previous reports regarding ILM-like effects when two cues were presented. It is also possible that dividing attention between two cues, two potential directions, or two potential line locations did not exceed attentional capacity of the participants. Alternatively, similarity of ratings regardless of how attention was allocated is consistent with the hypotheses that ILM-like effects do not involve attention (Blanco and Soto, 2009) or involve preattentive processes (von Grünau et al., 1996a; Kawahara and Yokosawa, 2001). However, it is doubtful the effect of pre-cue validity in Experiment 3 could be attributed to a lack of attention or to involvement of preattentive processes.

A second issue regarding the relationship between attention and ILM-like effects is whether such effects are accompanied by a shift of attention. Hamm and Klein (2002) suggested a shift of attention occurred subsequent to _flash_ILM and involved expansion of the focus of attention to incorporate the cued location and the entirety of the line that exhibited _flash_ILM. The lack of a Cue × Line interaction in Experiment 2 is consistent with limiting any shift of attention along the line to after illusory motion occurs, as had such shifts occurred prior to illusory motion, there should have been differences between ILM-like effects associated with a cue in the center (two possible directions of illusory motion) and ILM-like effects associated with cues on the left side or right side (one possible direction of illusory motion). However, an effect of pre-cue validity on ratings of relative velocity in Experiment 3 suggests a larger allocation of attention to the cued location prior to cue presentation can influence at least some aspects of ILM-like effects. It might be that SOAs of the cue and the line in previous studies of ILM-like effects were not long enough to allow consequences of a shift of attention to the cued location to be completed before the line was presented; if participants attend the cued location prior to appearance of the cue, and if there is no ambiguity regarding direction from the cue to the subsequent line, then participants might shift (or expand) attention in the anticipated direction of the line prior to presentation of the line (cf. Hubbard and Ruppel, 2013). This shift primes processing of (the near end of) the line, and so the near end of the line appears more quickly in perceptual awareness and relative velocity of illusory motion appears faster.

The role of attention in ILM-like effects has been debated, with suggestions that (a) attention is neither necessary (e.g., Blanco and Soto, 2009) nor sufficient (Downing and Treisman, 1997; Skottun, 2011) to produce ILM-like effects, (b) attention is sufficient to produce ILM-like effects (Bavelier et al., 2002), (c) exogenous attention but not endogenous attention is related to ILM-like effects (Christie and Klein, 2005; Christie, 2014; Ha et al., 2017), (d) endogenous attention can produce ILM-like effects (Schmidt, 2000; Bavelier et al., 2002), (e) endogenous attention can modulate but not produce ILM (Chica et al., 2008), (f) ILM-like effects facilitate shifts in visual attention (Crawford et al., 2006), (g) ILM-like effects are related to a shift of exogenous attention and a widening of the attentional “zoom lens” (Hamm and Klein, 2002), and (h) edge/surface counterchange rather than attentional tracking can produce ILM-like effects (Hock and Nichols, 2010). The different effects of attention in different studies, many of which used different methodologies, are consistent with Hamm’s (2017) suggestion that different ILM-like effects are (despite the phenomenological similarity of illusory motion) different effects caused by different mechanisms. Even so, the luminance cues in Experiments 1 and 2 involved exogenous attention, whereas the verbal pre-cues in Experiment 3 involved endogenous attention; thus, regardless of whether attention is generally necessary or sufficient for ILM-like effects, the data support previous claims that both exogenous attention and endogenous attention can influence ILM-like effects^3^.

The experiments reported here also addressed the relationship between relative velocity and relative strength of ILM-like effects. Intuitive notions that velocity reflects strength or that strength reflects velocity do not appear correct. In some cases, relative velocity and relative strength appeared positively correlated (e.g., effects of SOA). In other cases, relative velocity and relative strength appeared dissociated (e.g., effects of pre-cue validity). Curiously, if relative strength is measured indirectly by examining the extremity of ratings of direction, then relative strength increases with increases in SOA (Christie and Klein, 2005; Hubbard and Ruppel, 2011), whereas if relative strength is measured more directly by having participants rate perceived relative strength, then relative strength decreases with increases in SOA (Experiments 1, 2, and 3). von Grünau and Faubert (1994) found strength of illusory motion was related less to attributes of motion than to non-motion attributes that distinguished the line from the background (e.g., contrast, luminance, etc.). If relative strength of illusory motion is influenced by non-motion attributes, then it is not surprising that the relationship between relative strength and a variable related to motion such as relative velocity might appear inconsistent across experiments and measures. Indeed, given that perception of actual motion is influenced by similar non-motion attributes (e.g., Cavanagh et al., 1984; Stone and Thompson, 1992), it would not be surprising if perception of illusory motion is similarly influenced.

In Experiments 1, 2, and 3, ratings of velocity and ratings of strength decreased with increases in SOA between appearance of the cue and appearance of the line. Ratings of velocity generally decreased similarly across all SOAs, whereas ratings of strength appeared to decrease more between 50 and 250 ms than between 250 and 450 ms (indeed, the difference between 250 and 450 ms conditions was not significant in Experiment 3). The decrease in ratings of velocity with increases in SOA suggests that the processes that contribute to ILM-like effects linger for at least 450 ms; however, this is inconsistent with an attentional gradient hypothesis, as the attentional gradient fades more quickly (cf. Steinman et al., 1995). It is possible that ratings might have reflected some combination of TAM, PGM, and ILM, with different mechanisms contributing to ILM-like effects at different SOAs (cf. Hamm, 2017). It is also possible that decreases in ratings of velocity might reflect the slower pace of events within trials with longer SOAs rather than perceived velocity of the line *per se*. In other words, the speed of the display sequence (cue, SOA, line) might influence the perceived velocity of the line, such that slower display sequences result in slower perceived velocities. However, if the pace of the display sequence influenced velocity of illusory motion, then slower ratings of velocity should have occurred for longer SOAs even if a cue was not presented; such a pattern was not observed in Experiment 1.

The experiments reported here found that relative velocity and relative strength of ILM-like effects decreased with increases in SOA between presentation of the cue and presentation of the line. Despite this similarity, relative velocity and relative strength of illusory motion are at least partially dissociated (e.g., effects of pre-cue validity), and this is consistent with previous findings that relative strength of ILM-like effects can be influenced by non-motion attributes of the line. Whether illusory motion arises from one or two cues, or whether illusory motion might occur in only one direction or in one of two directions, does not influence relative velocity or relative strength of illusory motion. If attention is already at the cued location when the cue appears, relative velocity of illusory motion is increased. This latter finding is consistent with the hypothesis that as soon as a cue appears, attention is shifted in the direction of the anticipated line, and if attention is already at the cued location, a larger or more extensive shift in the direction of the anticipated line can occur before the line appears (thus leading to a faster entry into perceptual awareness and perception of a relatively faster velocity). Overall, ILM-like effects are primarily influenced by physical parameters such as SOA between the cue and the line and are less influenced by the allocation of attention and expectations regarding where the cue and the line will appear.

## Author Contributions

TH and SR contributed to the concept and design of the experiments. TH prepared the experimental programs and analyzed the data, and SR collected the data. TH drafted the paper, and SR provided critical feedback and revisions.

## Conflict of Interest Statement

The authors declare that the research was conducted in the absence of any commercial or financial relationships that could be construed as a potential conflict of interest.

## Footnotes

1 At the end of each trial in the strength block in each of Experiments 1, 2, and 3, participants also indicated the direction(s), if any, of perceived motion in that trial. Response options included “appeared all at once,” “from the left,” “from both the left end and the right end and inward toward the middle,” “from the middle outward toward both the left end and the right end,” and “from the right.” The responses were consistent with the predicted direction of ILM on each trial and served as a manipulation check that ILM occurred.

2 Many previous studies in which participants rated ILM-like effects on scales similar to those in the current experiments reported mean values and analyzed those values with parametric tests (e.g., Downing and Treisman, 1997; Christie and Klein, 2005; Chica et al., 2008; Ishigami et al., 2009). Even so, there have been suggestions that consideration of mean values and parametric tests are not appropriate for such Likert-type data (e.g., Jamieson, 2004); however, Norman (2010) has compellingly argued that parametric statistics can be used with Likert-type data.

3 Alternatively, exogenous and endogenous cues (corresponding to the square and the pre-cue, respectively, in Experiment 3) might orient different forms of attention (e.g., Briand and Klein, 1987), and it isn’t clear if an endogenous pre-cue might influence subsequent exogenous attention. A speculative possibility is that the dissociation between ratings of velocity and ratings of strength reflects different contributions of endogenous and exogenous forms of attention.
